# High level of inbreeding in final phase of 1000 Genomes Project

**DOI:** 10.1038/srep17453

**Published:** 2015-12-02

**Authors:** Steven Gazal, Mourad Sahbatou, Marie-Claude Babron, Emmanuelle Génin, Anne-Louise Leutenegger

**Affiliations:** 1 INSERM, IAME, UMR 1137, F-75018 Paris, France; 2 Plateforme de génomique constitutionnelle du GHU Nord, Assistance Publique des Hôpitaux de Paris (APHP), Hôpital Bichat, F-75018 Paris, France; 3 Fondation Jean Dausset CEPH, F-75010 Paris, France; 4 INSERM, Genetic variability and human diseases, UMR 946, F-75010 Paris, France; 5 Univ Paris-Diderot, UMR 946, Sorbonne Paris Cité, F-75010 Paris, France; 6 INSERM, UMR 1078, Génétique, Génomique fonctionnelle et Biotechnologies, F-29218 Brest, France; 7 Centre Hospitalier Régional Universitaire de Brest, F-29200 Brest, France

## Abstract

The 1000 Genomes Project provides a unique source of whole genome sequencing data for studies of human population genetics and human diseases. The last release of this project includes more than 2,500 sequenced individuals from 26 populations. Although relationships among individuals have been investigated in some of the populations, inbreeding has never been studied. In this article, we estimated the genomic inbreeding coefficient of each individual and found an unexpected high level of inbreeding in 1000 Genomes data: nearly a quarter of the individuals were inbred and around 4% of them had inbreeding coefficients similar or greater than the ones expected for first-cousin offspring. Inbred individuals were found in each of the 26 populations, with some populations showing proportions of inbred individuals above 50%. We also detected 227 previously unreported pairs of close relatives (up to and including first-cousins). Thus, we propose subsets of unrelated and outbred individuals, for use by the scientific community. In addition, because admixed populations are present in the 1000 Genomes Project, we performed simulations to study the robustness of inbreeding coefficient estimates in the presence of admixture. We found that our multi-point approach (FSuite) was quite robust to admixture, unlike single-point methods (PLINK).

The 1000 Genomes Project (TGP)^1^,^2^ provides a unique source of whole genome sequencing data, which aims at establishing a detailed catalog of human genetic variations. These data became an essential tool for a wide range of genetic studies, such as searching for natural selection in modern humans^3^,^4^,^5^, identifying patterns of linkage disequilibrium (LD), imputing millions of SNPs in genome-wide association studies data^6^, or filtering rare variants in monogenic disease studies^7^.

In June 2014, the third and final phase of this project consisted in more than 2,500 individuals from 26 populations (see Table 1 for the exhaustive list) divided into 5 super-populations: African (AFR), European (EUR), East Asian (EAS), South Asian (SAS) and Admixed American (AMR). Correct annotation of the genetic relationships in these reference samples is thus essential to guarantee unbiased results for further genetic studies^8^. However, while TGP individuals are described as unrelated and that relationships of the previous phases have been investigated by TGP consortium and others^2^,^9^, their inbreeding level is undocumented and could bias genotype and haplotype frequencies estimated on this panel. As we already reported inbreeding in HapMap phase III individuals^10^, some of whom are included in the TGP, we thus might expect inbreeding in this panel.

The goal of this article is to describe the inbreeding patterns in the 26 populations of the final phase of this panel by using the genotype data obtained from the sequencing. We applied our FSuite pipeline^11^, which estimates the genomic inbreeding coefficient *f* of individuals from their genotype data, and calculates the probability to be offspring of different mating types in order to infer the most likely relationship of the parents. As FSuite estimates are based on allele frequencies, it requires that the studied individuals come from a homogeneous population. However, some of the TGP populations are known to be admixed (AMR panel and ASW population), i.e. to have ancestry from different populations. In this case, the previous assumption is violated, which could bias FSuite *f* estimates. Indeed, it has been shown that single-point methods to estimate kinship and inbreeding coefficients are biased in presence of admixture in the population^12^,^13^ although this is less clear for multi-point methods^14^. For this reason, before estimating inbreeding in TGP populations, we investigated the accuracy of FSuite *f* estimates on admixed individuals by simulation.

## Results

### Overview of methods

The genome of an inbred individual is characterized by large regions homozygous-by-descent (HBD), i.e. regions where this individual has inherited two haplotypes identical-by-descent from a common ancestor. The genomic inbreeding coefficient *f* of an individual can thus be defined as the proportion of the genome that is HBD. Different approaches have been developed to estimate this coefficient from the genotype data of an individual without known genealogy, and can be classified in 2 main categories. First are single-point methods that rely only on the allele frequencies at each marker. Second are multi-point methods that use both allele frequencies and the fact that HBD genotypes come in blocks.

Our pipeline FSuite is a multi-point method, which requires the markers to be in minimal linkage disequilibrium (LD). FSuite therefore creates multiple random sparse marker maps (submaps) to minimize LD of the data while providing robust *f* estimations. Here, we ran FSuite on 100 submaps by selecting several random markers in each genomic regions delimited by recombination hotspots. This strategy optimizes the *f* estimation accuracy and the detection of inbred individuals^10^.

### FSuite performance in admixed samples

We investigated the accuracy of FSuite *f* estimates by simulating 100 replicates of a sample of 300 admixed individuals with different levels of inbreeding and different levels of European and African ancestry. FSuite multi-point estimates were first compared to the single-point estimates implemented in PLINK^15^. Each method was run with 4 sets of allele frequencies: frequencies estimated from European (CEU), African (YRI) and Asian (JPT/CHB) reference populations, and frequencies estimated on the sample (the FSuite default option, SAMPLE). We also ran the single-point method REAP^12^ on each simulated sample. REAP has been developed to estimate kinship and inbreeding coefficients in admixed samples using individual allele frequencies, i.e. allele frequencies weighted by the genomic proportion of the different ancestries estimated by Admixture^16^ software.

**Figure 1:**
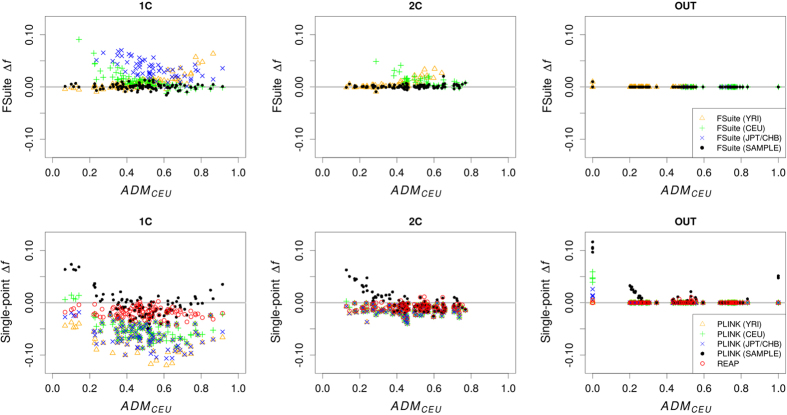
Accuracy of inbreeding estimators in simulated admixed samples. The differences between estimated and true *f* values (Δ*f*) and the genomic proportions of European ancestry (*ADM*_*CEU*_) were calculated on one random individual (1C, 2C and OUT) from 100 sample replicates (total 100 per mating type). Only FSuite estimates with Q >50 were plotted and single-point negative estimates (PLINK and REAP) were set to 0. Four sets of allele frequencies were used for FSuite and PLINK: European (CEU), African (YRI) and Asian (JPT/CHB) reference frequencies, and frequencies estimated on each sample (SAMPLE). REAP used individual allele frequencies. 1C = first-cousin offspring; 2C = second-cousin offspring; OUT = outbred individual.

Figure 1 shows the difference between FSuite estimates and true *f* value (Δ*f*) against the true genomic proportion of European ancestry (*ADM*_*CEU*_) of the individual, for first-cousin offspring (1C), second-cousin offspring (2C), and offspring of unrelated parents (OUT, as outbred) (see Figure S1 for more remote inbreeding). For FSuite, the quality of estimation was measured by a Q-score (see Methods). Based on simulations, estimation was defined as low quality if the Q-score was less than or equal to 50, this threshold being adequate to check whether the allelic frequencies were adapted for the *f* estimation (Table S1). Overall, FSuite estimates on admixed individuals were more robust than the ones from single-point methods, with smallest Δ*f* root mean square errors (RMSEs) whatever the set of allele frequencies (Table S2). Its estimates depended on *ADM*_*CEU*_ with CEU and YRI frequencies, showing that the *f* of admixed individuals in a homogeneous sample could be well estimated if less than 50% of their genome came from another population. Otherwise, the inbreeding coefficient *f* was biased or could not be estimated with FSuite, as can be seen with the JPT/CHB frequencies. Using frequencies estimated on the sample gave estimates with Δ*f* close to 0 whatever the admixture component of the individual and RMSEs below 5 × 10^−3^. On the opposite PLINK estimates had RMSEs always higher or equal to 13 × 10^−3^. Estimating individual allele frequency as done by REAP improved the single-point estimates (RMSE for OUT decreases from 26 × 10^−3^ with PLINK to 2 × 10^−3^), especially when *ADM*_*CEU*_ is far from 0.5. However, it did not significantly improve the accuracy of FSuite (Figure S2 and Table S2). We thus conclude that using allele frequencies estimated on an admixed sample, or on a homogeneous sample with some admixed individuals, gives robust estimates with multi-point methods, contrary to single-point methods, and thus does not require the estimation of ancestry proportions.

### Inbreeding estimation and detection on the last phase of 1000 Genomes project

Before applying FSuite on the TGP data, we ran the multi-point method RELPAIR^17^,^18^ on individual pairs from each population in order to detect unknown first or second degree relationships. Indeed, although the TGP final phase individuals have been described as unrelated based on pedigree information, no genetic analysis has been performed to uncover unknown relationships, as previously done for the phase I of this project. The choice of RELPAIR was motivated by our previous conclusion that multi-point methods are robust for admixed individuals. We detected 15 unreported relationships closer than first-cousins: 8 parent/offspring relationships (including one trio), 3 full-sibs, 1 half-sib, 3 avuncular relationships (Table S3). We thus excluded 14 individuals to estimate population allele frequencies.

**Figure 2:**
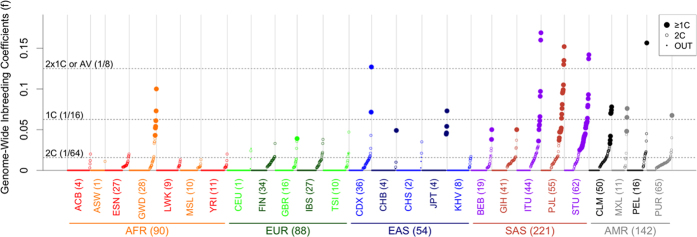
Inbreeding estimation and detection in TGP populations. Each point represents the *f* estimate for one individual. Large points represent the ones that are inferred as offspring of first-cousin (1C) or closest relationships, medium open points, the ones that are inferred as offspring of second-cousin offspring (2C), and small points, the ones that are inferred as outbred. Individuals were ordered in each population according to their *f* values. See Table 1 for the description of the different populations.

FSuite was then run independently on individuals from each of the 26 TGP populations totaling 2,504 individuals. Only 7 individuals coming from 3 populations (ASW, PEL and ITU) had a low-quality estimate (Q-score ≤ 50). Among the 2,497 remaining individuals, 595 individuals were inferred as inbred, which represents nearly a quarter of the panel (Tables 1 and S4, and Fig. 2). These individuals were mainly of SAS and AMR super-populations, with 45% and 41% of inbred individuals, respectively. We also found a high rate of inbred individuals in ESN (27%), GWD (25%), FIN (34%), IBS (25%) and CDX (39%). Only 6 populations out of the 26 have less than 5% of inbred individuals (ACB, ASW, CEU, CHB, CHS and JPT).

Most (501) of the 595 inbred individuals were inferred as 2C offspring and can be considered as descending from remote inbreeding. On the opposite, 94 individuals can be considered as descending from recent inbreeding, i.e. being offspring of first-cousin or closest relationships. These individuals belonged to 14 populations, including all SAS and AMR populations, but only one AFR population (6 from GWD) and one EUR population (one from GBR). Three-quarters of the inbred individuals came from 4 populations: ITU (10), PJL (22), STU (32) and CLM (8). Finally, note that GIH and PUR populations, that had a high proportion of inbred individuals (40% and 64%, respectively), had only one individual who exhibited recent inbreeding. Of interest, the most inbred European population is the Finnish with one third of individuals detected as remote inbreeding (34% as 2C and maximum *f* equals to 0.035). This is in accordance with the population history of the Finns: a small number of founders and very little immigration^19^.

### Unrelated and outbred panel of the last phase of 1000 Genomes project

From these findings, we propose two different panels of unrelated and outbred individuals to TGP users, such as previously proposed for HapMap III panel^8^,^10^. First, for both panels, we removed 7 individuals for quality reasons (Q-score ≤ 50). Then, for the first panel, labeled TGP2457, we removed the 14 individuals involved in first and second degree relationships inferred by RELPAIR, and 26 individuals inferred as avuncular offspring (AV) or double first-cousin offspring (2 × 1C) by FSuite. Finally for the second panel, labeled TGP2261, we removed individuals from 227 relationships up to first-cousins detected by RELPAIR (see Table S3), and the 94 individuals that have been inferred as first-cousin offspring or closer by FSuite. These filters mainly reduced the number of individuals in STU, PJL, ASW and LWK populations, with a sample size decrease of 35%, 31%, 26% and 23%, respectively (Table S5). These 2 lists are provided in Table S4.

## Discussion

In conclusion, we have shown that multi-point approaches provide reliable estimates of the genomic inbreeding coefficient *f* even when there are some admixed individuals in the studied population. This is not the case for single-point approaches that require reliable estimates of both the allele frequencies in the parent populations and the ancestry proportions. These estimates being difficult to obtain, there is a real advantage of multi-point approaches over single-point methods. This finding is in accordance with the results of Thompson and Kuhner^14^ for genomic segments shared identical-by-descent between pairs of individuals.

On the final phase (Phase III) of the 1000 Genome Project, we found that nearly a quarter of the individuals in this panel were inbred and that around 4% of them had inbreeding coefficients similar or greater than the ones expected for first-cousin offspring. This level of inbreeding was unexpectedly high, and is much higher than the 4% of inbred individuals that we detected on HapMap III^10^. This difference has 2 main explanations. First, the most inbred TGP populations were not present in HapMap III and some of them are known to have high levels of consanguineous marriages, such as Indian and Pakistani populations^20^. Second, in this study we used FSuite with submaps delimited by recombination hotspots, rather than arbitrary distances (0.5 cM). Selecting markers based on recombination hotspots improves inbreeding detection, in particular for relationships deeper than second-cousin^10^. Among the 756 individuals that are both in HapMap III and TGP, submaps delimited by recombination hotspots allowed the detection of 44 additional inbred individuals (Figure S3), especially in the GIH population that has a high level of recent inbreeding (26 individuals).

Finally, we also detected pairs of close relatives (up to first-cousin) not reported from pedigree information. We thus propose two subsets of unrelated and outbred individuals. The first one, labeled TGP 2457, removes relatedness of first and second degree, similarly to what has been performed previously for HGDP-CEPH panel^21^ and HapMap III panel^8^, and in addition it removes offspring of relationships of the same degree. As we observed a lot of first-cousin relationships and first-cousin offspring (especially in AFR and SAS populations), it seemed important to us to also remove them and to create the TGP2261 panel. We would hence recommend this latter panel to the scientific community.

## Methods

### Inbreeding estimation and detection with FSuite

FSuite is a pipeline that allows running FEstim software^22^ on multiple submaps minimizing LD of the data^23^. To model the HBD process of an individual, FEstim uses a hidden Markov model (HMM) in which the emission probabilities depend on allele frequencies. Its Markov chain depends on two parameters *f* and *a*, defining *f* as the individual inbreeding coefficient and 1/(*a*(1 − *f*)) as the expected length of HBD segments (here cM), that are estimated by maximum likelihood. FSuite estimates *f* as the median value of the estimates obtained on the different submaps after removing the ones with *a* > 1, and thus calculates a quality Q-score of each estimate as the number of submaps that have not been removed. FSuite also uses FEstim likelihood computed on the different submaps to estimate whether an individual is inbred or not by a likelihood ratio test testing if *f* is significantly different from 0.001 (extensive simulations have shown that this value provides accurate detection of inbred individuals^10^), and to calculate probabilities for each individual to be offspring of different mating types. Types of offspring considered by FSuite are avuncular offspring (AV), double first-cousin offspring (2 × 1C), first-cousin offspring (1C), second-cousin offspring (2C), and offspring of unrelated parents (OUT, as outbred).

To improve the accuracy of inbreeding detection, we created 100 submaps by selecting several random markers in each genomic regions delimited by HapMap II recombination hotspots^24^,^25^ having recombination intensity >10 cM/Mb, as recommended in Gazal *et al*.^10^. Each submap contained 14,322 SNPs in the simulation study, and 12,064 SNPs in the application to TGP data. The most likely mating type of an individual was defined as the mating type with the highest probability. Here, an individual was reported as inbred if both his/her *f* was significantly different from 0.001 (p-value < 0.05) and if he/she was inferred as AV, 2 × 1C, 1C or 2C.

### Admixture simulation study

In order to investigate the accuracy of FSuite estimates in admixed populations, we simulated samples in which individuals had different proportions of European and African ancestry. For this purpose, we used the same simulation process as in Gazal *et al*.^10^. Briefly, 100 replicates of samples of 300 individuals were simulated with different level of inbreeding: 6 individuals were offspring of 1C, 6 offspring of 2C, 18 offspring of third-cousin (3C), 30 offspring of fourth-cousin (4C) and 240 offspring of unrelated individuals OUT. For each individual, we first simulated the recombination process on the genealogy. To have realistic LD patterns, we used haplotypes estimated from HapMap III populations as reference.

Here, we used 232 and 226 haplotypes of CEU and YRI, respectively, with 987,221 SNPs coming from the Illumina Human 1M chip or the Affymetrix v6.0 chip. To simulate an individual genome, we first assigned randomly to each pedigree founder a haplotype origin (CEU or YRI). For example, a 1C offspring will have randomly between 0 and 8 pedigree founders with a CEU origin. Then, according to this haplotype origin, HapMap reference haplotypes were randomly drawn without replacement for each chromosome and were assigned to pedigree founders to construct the genotype data of the individual.

Pedigree founders were used to detect the true HBD segments of an individual, and the haplotypes of CEU and YRI origin. The true inbreeding coefficient of an individual (*f*_*true*_) was calculated by dividing the genome length in cM that is HBD by the total genome length. The genome length was obtained by adding the genetic distance between the first and the last marker on each autosome. The CEU admixture component (*ADM*_*CEU*_) of an individual was calculated as the proportion of genome in cM with CEU reference haplotypes.

To estimate inbreeding in these simulated admixed samples, we first created 4 different sets of allele frequencies. First, we used CEU allele frequencies (estimated on the 232 reference haplotypes) and YRI (estimated on the 226 ones), to investigate inbreeding estimation of an admixed individual in a homogeneous sample. Then, to investigate the impact of inappropriate allele frequencies, we used 340 HapMap III haplotypes coming from JPT and CHB populations (JPT/CHB). Finally, we estimated allele frequencies as it is done by default, i.e. on the 300 individuals of the sample.

FSuite was thus run on each replicate with these 4 different sets of allele frequencies. To compare the benefit of using multi-point methods, such as the one implemented in FSuite, to single-point ones, we first estimated *f* in each replicate through the single-point method implemented in PLINK version 1.90b2b. Then, to take into account the admixture of the individuals, we also ran single-point method implemented in REAP version 1.2 on each simulated sample. Ancestry proportion of each individual and allele frequencies of each of the ancestral populations were estimated by running software Admixture version 1.23 on each sample; LD within each sample was removed as advised in the Admixture documentation (PLINK pruning option–indep-pairwise 50 10 0.1), and the number of ancestral populations was set to K = 2. All PLINK and REAP negative estimates were set to 0. The performances of the different estimators were compared on the 5 different types of offspring (1-4C and OUT) by randomly drawing one individual of each type from each replicate with *f*_true_ > 0. Accuracy of the different estimators was assessed by measuring the difference between the estimated *f* and their corresponding *f*_true_ (Δ*f*), and by computing its root mean square error (RMSE) as described previsouly^10^.

Finally, to investigate the benefits of using individual allele frequencies with FSuite, we computed for each individual their theoretical individual allele frequencies, obtained by weighting CEU allele frequencies and the YRI allele frequencies by their true CEU (*ADM*_*CEU*_) and YRI admixture components, respectively.

### 1000 Genomes Phase III data quality control

The final release of the Phase III consists in sequencing data of 2,535 individuals representing 26 populations divided into 5 super populations. We downloaded via ftp the vcf file of the final variant set (release v5 20130502), from which 31 related individuals have been removed based on pedigree information and on the results of the genetic analysis that has been performed to uncover unknown relationships of the phase I of this project^2^. We used PLINK to extract biallelic SNPs located on autosomes from the more than 81 million variants available. Only the most informative SNPs were kept, i.e. with minor allele frequency ≥0.05 in each of the 26 populations. Finally, SNPs departing from Hardy-Weinberg equilibrium (p < 10^−5^ in at least one population) were excluded. After these different filters, 3,033,793 SNPs were retained. The genetic positions of these SNPs were extrapolated from HapMap II genetic map.

We then ran RELPAIR version 2.0.1 on every pair of individuals from each population in order to remove unknown first or second-degree relationships to estimate population allele frequencies. The submap approach used in FSuite was extended to RELPAIR. We created 100 submaps using recombination hotspots having recombination intensity >13 cM/Mb, this threshold allowing selecting less than the 9,999 SNPs allowed by RELPAIR (9,372 SNPs) while minimizing LD. For each pair, the relationship inferred the largest number of times on the 100 submaps was reported. Note that none of the detected relationships was previously reported by the phase I of this project.

## Additional Information

**How to cite this article**: Gazal, S. *et al*. High level of inbreeding in final phase of 1000 Genomes Project. *Sci. Rep.* **5**, 17453; doi: 10.1038/srep17453 (2015).

## Supplementary Material

Supplementary Information

Supplementary Table S3

Supplementary Table S4

## Figures and Tables

**Table 1:** Inbreeding detection in TGP populations.

	**Total**	**AV**	**2 × 1C**	**1C**	**2C**	**Total inbred**
**African (AFR)**	**660**	**—**	**—**	**6**	**84**	**90 (14%)**
African Caribbean in Barbados (ACB)*	96	**—**	**—**	**—**	4	4 (4%)
African Ancestry in Southwest United States (ASW)*	60	**—**	**—**	**—**	1	1 (2%)
Esan in Nigeria (ESN)	99	**—**	**—**	**—**	27	27 (27%)
Gambian in Western Division, The Gambia (GWD)	113	**—**	**—**	6	22	28 (25%)
Luhya in Webuye, Kenya (LWK)	99	**—**	**—**	**—**	9	9 (9%)
Mende in Sierra Leone (MSL)	85	**—**	**—**	**—**	10	10 (12%)
Yoruba in Ibadan, Nigeria (YRI)	108	**—**	**—**	**—**	11	11 (10%)
**European (EUR)**	**503**	**—**	**—**	**1**	**87**	**88 (17%)**
Utah residents with European ancestry (CEU)	99	**—**	**—**	**—**	1	1 (1%)
Finnish in Finland (FIN)	99	**—**	**—**	**—**	34	34 (34%)
British in England and Scotland (GBR)	91	**—**	**—**	1	15	16 (18%)
Iberian populations in Spain (IBS)	107	**—**	**—**	**—**	27	27 (25%)
Toscani in Italy (TSI)	107	**—**	**—**	**—**	10	10 (9%)
**East Asian (EAS)**	**504**	**—**	**1**	**6**	**47**	**54 (11%)**
Chinese Dai in Xishuangbanna, China (CDX)	93	**—**	1	1	34	36 (39%)
Han Chinese in Bejing, China (CHB)	103	**—**	**—**	1	3	4 (4%)
Southern Han Chinese, China (CHS)	105	**—**	**—**	**—**	2	2 (2%)
Japanese in Tokyo, Japan (JPT)	104	**—**	**—**	4	**—**	4 (4%)
Kinh in Ho Chi Minh City, Vietnam (KHV)	99	**—**	**—**	**—**	8	8 (8%)
**South Asian (SAS)**	**487**	**—**	**23**	**44**	**154**	**221 (45%)**
Bengali in Bangladesh (BEB)	86	**—**	**—**	2	17	19 (22%)
Gujarati Indian in Houston, Texas (GIH)	103	**—**	**—**	1	40	41 (40%)
Indian Telugu in the United Kingdom (ITU)	100	**—**	4	6	34	44 (44%)
Punjabi in Lahore, Pakistan (PJL)	96	**—**	9	13	33	55 (57%)
Sri Lankan Tamil in the United Kingdom (STU)	102	**—**	10	22	30	62 (61%)
**Admixed American (AMR)**	**343**	**1**	**1**	**11**	**129**	**142 (41%)**
Colombian in Medellin, Colombia (CLM)	94	**—**	**—**	8	42	50 (53%)
Mexican Ancestry in Los Angeles, California (MXL)	64	**—**	1	2	8	11 (17%)
Peruvian in Lima, Peru (PEL)	81	1	**—**	**—**	15	16 (20%)
Puerto Rican in Puerto Rico (PUR)	104	**—**	**—**	1	64	65 (63%)
**TOTAL**	**2497**	**1**	**25**	**68**	**501**	**595 (24%)**

1 ASW, 2 ITU and 4 PEL of the 2,504 initial individuals have been removed due to Q-score ≤ 50. AV = avuncular offspring; 2 × 1C = double first-cousin offspring; 1C = first-cousin offspring; 2C = second-cousin offspring.

^*^These populations should be considered as Admixed African.
