# Supplementary material for: High level of inbreeding in final phase of 1000 Genomes Project
Source: Sci Rep. 2015 Dec 2;5:17453. doi: 10.1038/srep17453 (PMC4667178; doi:10.1038/srep17453)
Supplement: Supplementary Information [file srep17453-s1.doc]

# Supplementary information for “High level of inbreeding in final phase of 1000 Genomes Project”

Steven Gazal, Mourad Sahbatou, Marie-Claude Babron, Emmanuelle Génin, Anne-Louise Leutenegger

# Supplementary Figures

**
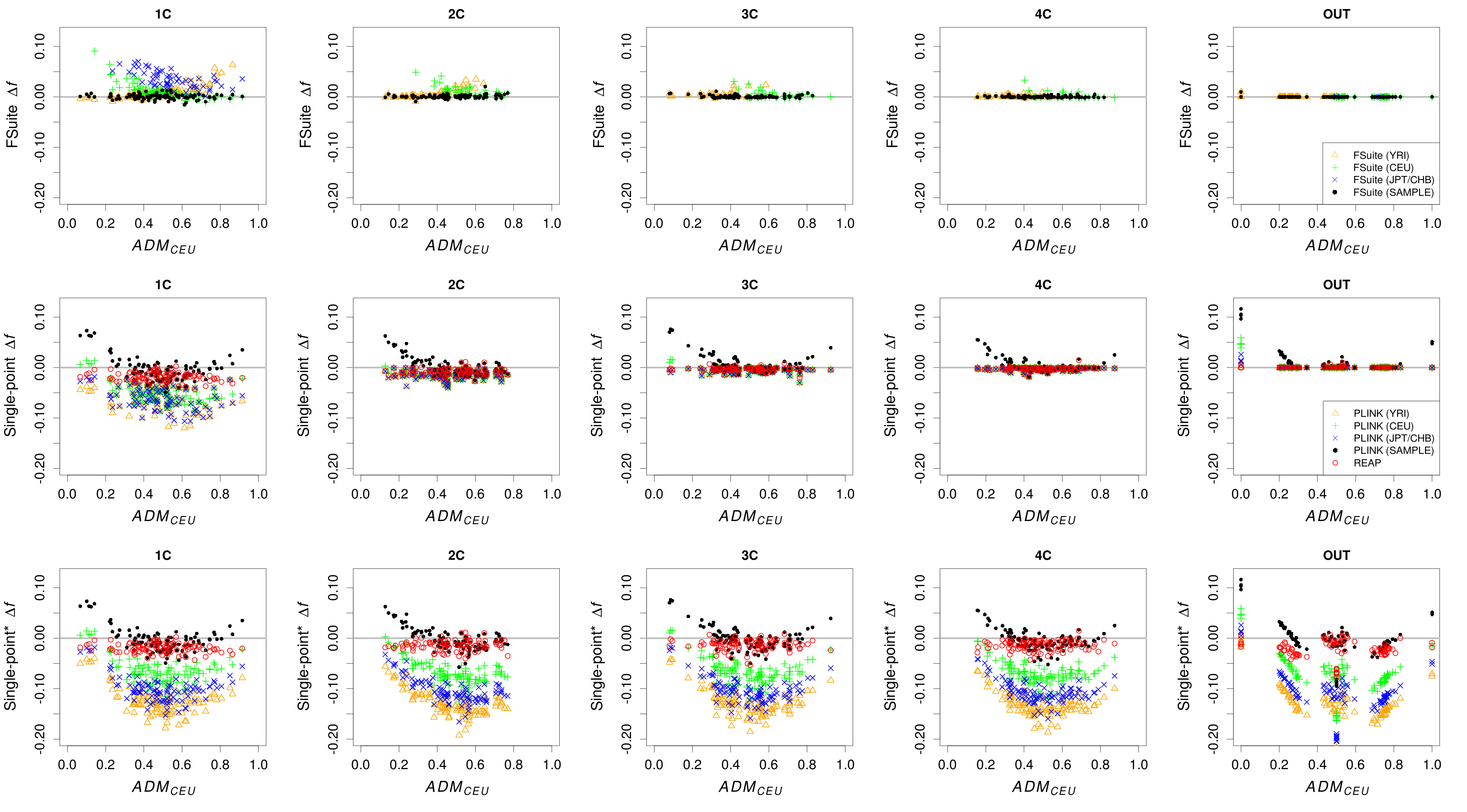
**

**Figure S1: Accuracy of inbreeding estimators in admixed simulated samples.** Difference between estimated and true *f* values (Δ*f*) and the genomic proportion of European ancestry (*ADM_CEU_*) of the individual were calculated on one random individual of each type from each replicate (total 100). Only FSuite estimates with Q > 50 were plotted. Second row (Single-point) sets negative estimates to 0 while third row (Single-point*) keeps negative estimates. Third row legend is the same as the second row. Four sets of allele frequencies were used for FSuite and PLINK: European (CEU), African (YRI) and Asian (JPT/CHB) reference frequencies, and frequencies estimated each sample (SAMPLE). REAP estimated individual allele frequencies. 1C = first-cousin offspring; 2C = second-cousin offspring; 3C = third-cousin offspring; 4C = fourth-cousin offspring; OUT = outbred individual.


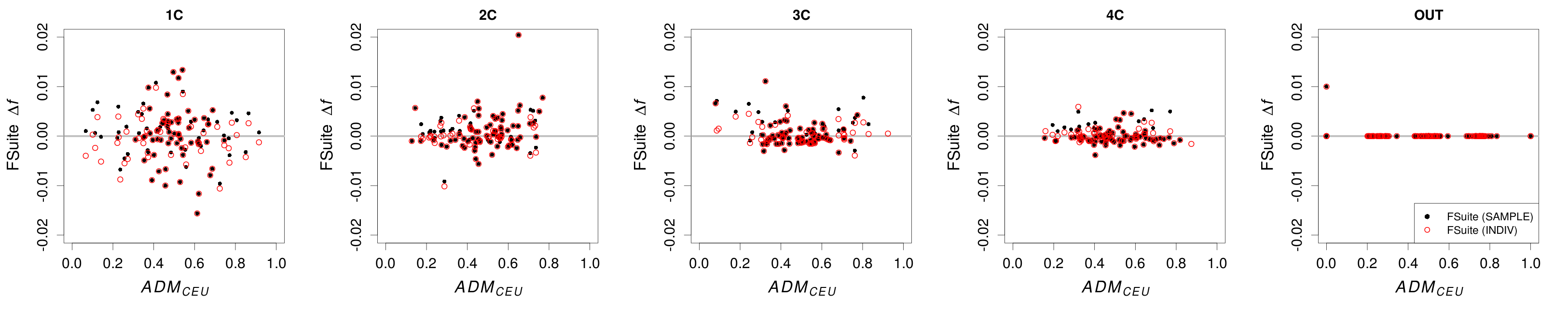


**Figure S2: Benefits of using individual allele frequencies with FSuite.** Difference between estimated and true *f* values (Δ*f*) and the genomic proportion of European ancestry (*ADM_CEU_*) of the individual were calculated on one random individual of each type from each replicate (total 100). Only FSuite estimates with Q > 50 were plotted. Two sets of allele frequencies were used: estimated on the sample (SAMPLE), and theoretical individual allele frequencies (INDIV), obtained by weighting CEU allele frequencies and the YRI allele frequencies by their true CEU and YRI admixture components, respectively.


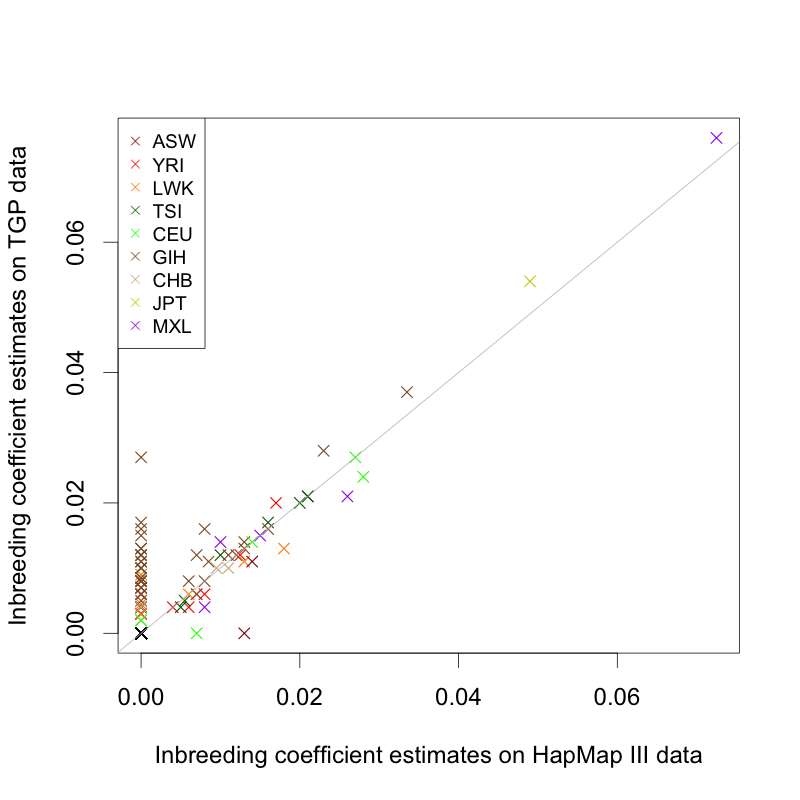


**Figure S3: Comparison of TGP *f* estimates with their previously published estimation on HapMap III data.** Inbreeding coefficients of TGP data were estimated using FSuite with submaps delimited by recombination hotspots. Inbreeding coefficients of HapMap III data were estimated using FSuite with submaps taking one marker per 0.5 cM. On the 756 individuals that are both in HapMap III and TGP, 669 have an *f* estimated at 0 on both datasets (these individuals are concentrated on the black cross in the figure), 41 have an *f* different of 0 on both datasets (correlation = 0.98), 44 have an have an *f* different of 0 only on TGP data (including 26 GIH), and 2 have an have an *f* different of 0 only on HapMap III data.

# Supplementary Tables

| **Q** | **YRI** | | | | | **CEU** | | | | | **JPT/CHB** | | | | | **SAMPLE** | | | | |
| --- | --- | --- | --- | --- | --- | --- | --- | --- | --- | --- | --- | --- | --- | --- | --- | --- | --- | --- | --- | --- |
|  | **1C** | **2C** | **3C** | **4C** | **OUT** | **1C** | **2C** | **3C** | **4C** | **OUT** | **1C** | **2C** | **3C** | **4C** | **OUT** | **1C** | **2C** | **3C** | **4C** | **OUT** |
| **0** | 2 | 24 | 30 | 23 | 8 | 3 | 29 | 30 | 26 | 7 | 24 | 93 | 80 | 66 | 26 | - | - | - | - | - |
| **]0-5]** | 1 | 8 | 11 | 14 | 16 | - | 4 | 5 | 11 | 10 | 6 | 6 | 11 | 19 | 29 | - | - | - | - | - |
| **]5-50]** | 3 | 11 | 22 | 23 | 25 | 3 | 13 | 20 | 30 | 26 | 8 | 1 | 9 | 15 | 31 | - | - | 2 | 2 | - |
| **]50-95[** | - | 12 | 20 | 31 | 10 | 4 | 9 | 29 | 25 | 14 | 8 | - | - | - | 14 | - | 2 | 9 | 10 | 4 |
| **[95-100]** | 94 | 45 | 17 | 9 | 41 | 90 | 45 | 16 | 8 | 43 | 54 | - | - | - | - | 100 | 98 | 89 | 88 | 96 |

**Table S1: Quality of FSuite (Q) with different allele frequencies on admixed individuals.**

| **Method** | **Allele frequencies** | **1C** | **2C** | **3C** | **4C** | **OUT** |
| --- | --- | --- | --- | --- | --- | --- |
| **FSuite** | **YRI** | 15.27 | 11.01 | 7.77 | 2.81 | 1.4 |
|  | **CEU** | 18.42 | 14.22 | 8.07 | 7.34 | 0.00 |
|  | **JPT/CHB** | 38.50 | - | - | - | 0.00 |
|  | **SAMPLE** | 5.07 | 3.50 | 2.63 | 1.66 | 1.00 |
|  | **Theoretical individual allele frequencies** | 5.05 | 3.43 | 2.19 | 1.43 | 1.00 |
| **PLINK** | **YRI** | 66.31 | 16.74 | 7.17 | 4.33 | 0.34 |
|  | **CEU** | 53.91 | 16.21 | 7.48 | 4.33 | 10.79 |
|  | **JPT/CHB** | 63.72 | 16.74 | 7.17 | 4.33 | 3.52 |
|  | **SAMPLE** | 23.33 | 17.18 | 17.16 | 12.98 | 26.13 |
| **REAP** | **Estimated individual allele frequencies** | 21.13 | 11.81 | 5.45 | 4.28 | 1.54 |

**Table S2: Root mean square error (RMSE) for different estimators.** Numbers are per 1,000 (10^-3^). Only FSuite estimates with Q > 50 were used and single-point negative estimates (PLINK and REAP) were set to 0. Different sets of allele frequencies were used for FSuite and PLINK: European (CEU), African (YRI) and Asian (JPT/CHB) reference frequencies, and frequencies estimated on each sample (SAMPLE). Theoretical individual allele frequencies where also used with FSuite. REAP used estimated individual allele frequencies.

See supplemental excel file Table S3.

**Table S3: RELPAIR results**

See supplemental excel file Table S4.

**Table S4: FSuite results**

|  | **Final phase** | **TGP2457** | **TGP2261** |
| --- | --- | --- | --- |
| **African (AFR)** | **660** | **651** | **577** |
| African Caribbean in Barbados (ACB)* | 96 | 95 | 92 |
| African Ancestry in Southwest United States (ASW) * | 60 | 54 | 45 |
| Esan in Nigeria (ESN) | 99 | 99 | 86 |
| Gambian in Western Division, The Gambia (GWD) | 113 | 113 | 96 |
| Luhya in Webuye, Kenya (LWK) | 99 | 97 | 76 |
| Mende in Sierra Leone (MSL) | 85 | 85 | 75 |
| Yoruba in Ibadan, Nigeria (YRI) | 108 | 108 | 107 |
| **European (EUR)** | **503** | **503** | **489** |
| Utah residents with European ancestry (CEU) | 99 | 99 | 94 |
| Finnish in Finland (FIN) | 99 | 99 | 99 |
| British in England and Scotland (GBR) | 91 | 91 | 85 |
| Iberian populations in Spain (IBS) | 107 | 107 | 107 |
| Toscani in Italy (TSI) | 107 | 107 | 104 |
| **East Asian (EAS)** | **504** | **502** | **481** |
| Chinese Dai in Xishuangbanna, China (CDX) | 93 | 92 | 82 |
| Han Chinese in Bejing, China (CHB) | 103 | 103 | 102 |
| Southern Han Chinese, China (CHS) | 105 | 104 | 99 |
| Japanese in Tokyo, Japan (JPT) | 104 | 104 | 100 |
| Kinh in Ho Chi Minh City, Vietnam (KHV) | 99 | 99 | 98 |
| **South Asian (SAS)** | **487** | **460** | **397** |
| Bengali in Bangladesh (BEB) | 86 | 86 | 83 |
| Gujarati Indian in Houston, Texas (GIH) | 103 | 101 | 96 |
| Indian Telugu in the United Kingdom (ITU) | 100 | 96 | 86 |
| Punjabi in Lahore, Pakistan (PJL) | 96 | 87 | 66 |
| Sri Lankan Tamil in the United Kingdom (STU) | 102 | 90 | 66 |
| **Admixed American (AMR)** | **343** | **341** | **317** |
| Colombian in Medellin, Colombia (CLM) | 94 | 94 | 80 |
| Mexican Ancestry in Los Angeles, California (MXL) | 64 | 63 | 58 |
| Peruvian in Lima, Peru (PEL) | 81 | 80 | 79 |
| Puerto Rican in Puerto Rico (PUR) | 104 | 104 | 100 |
| **TOTAL** | **2497** | **2457** | **2261** |

*These populations should be considered as Admixed African

**Table S5: Description of panels TGP2457 and TGP2261.** Panel TGP2457 removed 14 individuals involved in 1^st^ and 2^nd^ degree relationships by RELPAIR, 26 individuals inferred as avuncular offspring (AV) or double first-cousin offspring (2x1C) by FSuite, and the 7 individuals with a low Q-score in the FSuite analysis. Panel TGP2261 removed individuals from the 227 relationships detected by RELPAIR, 94 individuals that have been inferred as offspring of first-cousins or closer relationships by FSuite, and the 7 individuals with a low Q-score in the FSuite analysis.
